# Survival bias and declining mortality risk in long‐term COVID‐19 studies

**DOI:** 10.1111/irv.70247

**Published:** 2026-03-06

**Authors:** Wiessam Abu Ahmad, Ronen Arbel

**Affiliations:** ^1^ Braun School of Public Health Hebrew University‐Hadassah Jerusalem Israel; ^2^ Branch of Planning and Strategy Clalit Health Services Tel Aviv Israel; ^3^ Community Medical Services Division Clalit Health Services Tel‐Aviv Israel; ^4^ Maximizing Health Outcomes Research Lab Sapir College Sderot Israel

The declining mortality hazard ratios observed over 3 years in COVID‐19 survivors may reflect survival bias rather than genuine risk convergence. Quantifying acute‐phase mortality risk through propensity scoring or landmark stratification could distinguish survivor enrichment effects from true clinical improvement in long‐term outcome trajectories.

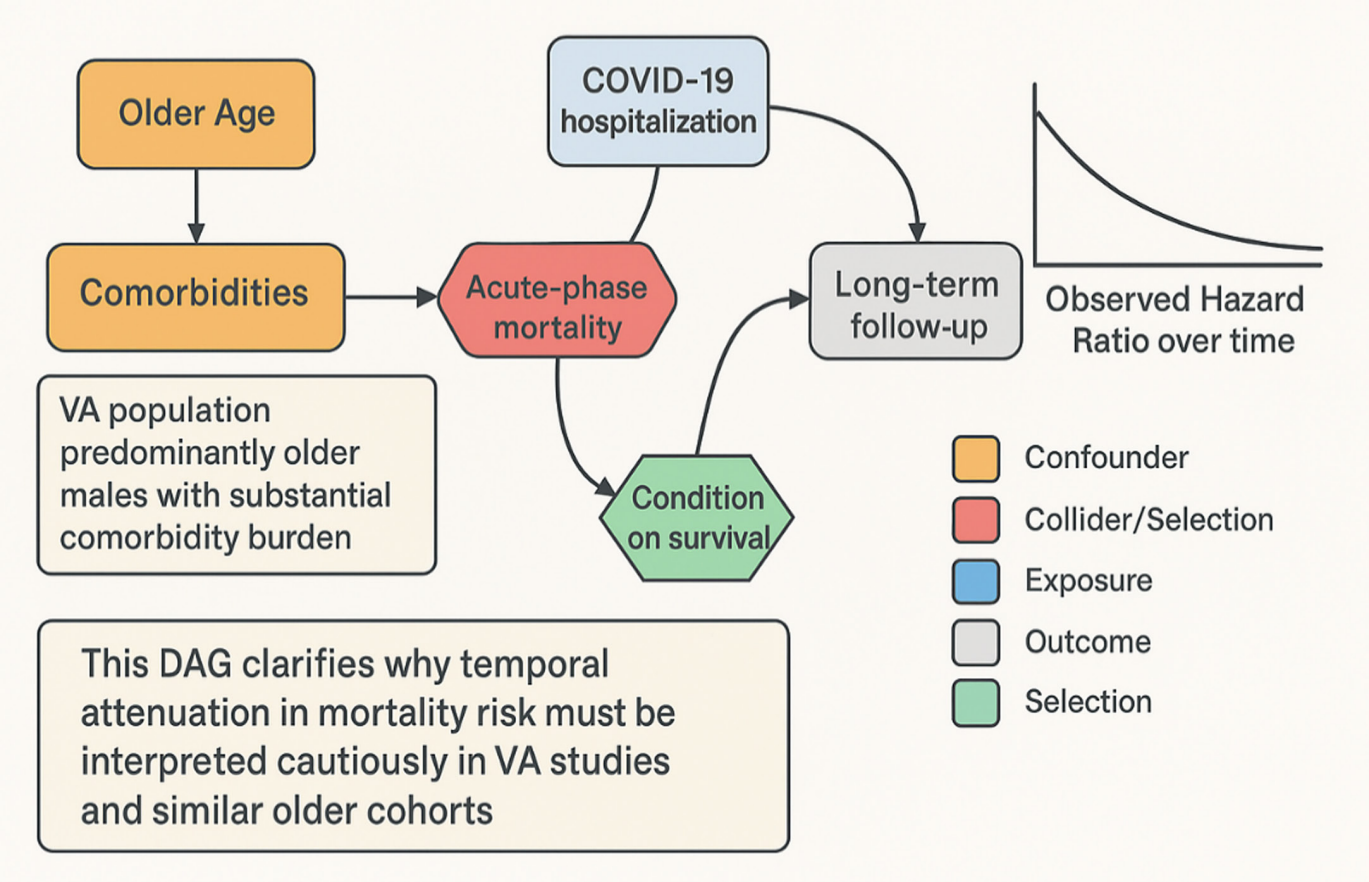

The 3‐year follow‐up analysis by Cai and colleagues [1] extends our understanding of long‐term COVID‐19 sequelae. However, the progressive attenuation of mortality risk in hospitalized COVID‐19, from IRR 3.17 (95% Confidence Interval [CI]: 3.00‐3.33) in year 1 to IRR 1.29 (1.19‐1.40) in year 3, may reflect survival bias rather than genuine risk convergence. This systematic selection effect occurs because individuals surviving to later follow‐up represent an increasingly enriched cohort of inherently healthier individuals with better organ reserve and less severe acute‐phase injury, independent of true clinical improvement.

This concern is particularly relevant for hospitalized COVID‐19 survivors, where baseline heterogeneity in organ dysfunction severity cannot be fully captured by standard covariates. Substantial early mortality concentrated among vulnerable individuals, with acute‐phase excess mortality highest in the first 90 days (HR = 6.25) [2], creates conditions for collider stratification bias [3]. Because survival is conditioned on not dying early, the remaining cohort becomes progressively healthier, falsely attenuating observed risks through survivor enrichment rather than genuine disease resolution.

The causal structure reflects collider bias: older age and comorbidities predict both acute‐phase and long‐term mortality. Many high‐risk patients die acutely, leaving only healthier survivors in long‐term follow‐up. The apparent reduction in mortality risk may therefore indicate selective survival of low‐risk phenotypes rather than true clinical improvement.

To evaluate whether temporal IRR attenuation reflects genuine convergence or survivor enrichment, we recommend two analytical approaches. First, quantify each individual's predicted probability of acute‐phase mortality based on baseline characteristics and weight analyses to account for heterogeneity in acute‐phase survival risk. Second, conduct stratified landmark analyses according to predicted acute‐phase mortality risk strata. If attenuation reflects true recovery, within‐stratum IRRs should remain stable or continue to decline. If collider bias drives the pattern, within‐stratum estimates should reveal persistent elevated and stable hazards at later follow‐up periods, with apparent overall attenuation reflecting compositional changes rather than genuine improvement.

This survival selection mechanism is not unique to Cai et al.'s cohort but is likely to affect most longitudinal COVID‐19 and infectious disease studies that follow survivors over time without explicitly modeling acute‐phase mortality and conditioning structure. Any design that restricts analysis to individuals who survive an initial high‐risk period, particularly when baseline vulnerability is unevenly distributed across age, comorbidity, or socioeconomic strata, is susceptible to the same progressive enrichment of lower‐risk survivors and spurious attenuation of hazards. Consequently, similar patterns of “waning” excess risk reported in other post‐acute COVID‐19 and hospitalization cohorts should be interpreted cautiously and informing post‐acute care strategies.

## Author Contributions

**Wiessam Abu Ahmad:** conceptualization, investigation, writing – original draft, methodology, validation, visualization. **Ronen Arbel:** methodology, supervision, writing – review and editing.

## Funding

The authors have nothing to report.

## Conflicts of Interest

The authors declare no conflicts of interest.

## Data Availability

Data sharing is not applicable to this article as no datasets were generated or analyzed during the current study.
